# KDF1, a Novel Tumor Suppressor in Clear Cell Renal Cell Carcinoma

**DOI:** 10.3389/fonc.2021.686678

**Published:** 2021-05-31

**Authors:** Jing-min Zheng, Mei-fu Gan, Hong-yuan Yu, Lu-xia Ye, Qing-xin Yu, Yu-hui Xia, Han-xi Zhou, Jia-qian Bao, Yi-qing Guo

**Affiliations:** ^1^ Department of Urology, Taizhou Hospital, Wenzhou Medical University, Linhai, China; ^2^ Department of Pathology, Taizhou Hospital, Wenzhou Medical University, Linhai, China

**Keywords:** KDF1, clear cell renal cell carcinoma, tumor grade, tumor suppressor, prognosis

## Abstract

KDF1 has been identified as a key regulator of epidermal proliferation and differentiation, but it is unknown whether KDF1 is involved in the pathogenesis of malignancy. No study has reported the expression and function of KDF1 in renal cancer. To explore the pathologic significance of KDF1 in clear cell renal cell carcinoma (ccRCC), the expression level of KDF1 protein in the tumor tissue of ccRCC patients was examined by immunohistochemistry and Western blot while the expression level of KDF1 mRNA was analyzed by using the data from TCGA database. In vitro cell experiments and allogeneic tumor transplantation tests were performed to determine the effects of altered KDF1 expression on the phenotype of ccRCC cells. Both the KDF1 mRNA and protein were found to be decreasingly expressed in the tumor tissue of ccRCC patients when compared with the adjacent non-tumor control tissue. The expression level of KDF1 in the tumor tissue was found to correlate negatively with the tumor grade. Patients with higher KDF1 in the tumor tissue were found to have longer overall survival and disease-specific survival time. KDF1 was shown to be an independent factor influencing the disease-specific survival of the ccRCC patients. Overexpression of KDF1 was found to inhibit the proliferation, migration and invasion of ccRCC cells, which could be reversed by decreasing the expression of KDF1 again. ccRCC cells with KDF1 overexpression were found to produce smaller transgrafted tumors. These results support the idea that KDF1 is involved in ccRCC and may function as a tumor suppressor.

## Introduction

Kidney cancer is one of the ten most commonly diagnosed cancers worldwide and the most lethal of the genitourinary tumors. More than 175,000 people died of kidney cancer in 2018 and the incidence is still rising (1). Clear cell renal cell carcinoma (ccRCC) is a major histological subtype of kidney cancer (accounting for 60-80% of the disease) and the most malignant one of the disease (1). Treatment of ccRCC is not very effective due to its resistance to commonly used chemotherapy and radiation (2). Early surgical resection is still the preferred therapy. However, relapse and metastasis are common even in patients with localized disease after radical nephrectomy (3). Targeted therapy has been introduced to treat ccRCC and has partially extended patients’ survival time. However, none of the therapies result in a durable response (4, 5). Thus, there is an urgent need to further explore the mechanism of ccRCC, find new diagnostic and/or prognostic markers, and develop novel therapies to improve the prognosis of the disease.

In 2013, Lee et al. reported a recessive mutant mouse with a short snout and short limbs, which they designated as shorthand (*shd*) and was further proved to be caused by a mutation in the Keratinocyte Differentiation Factor 1 (KDF1) gene, in an N-ethyl-N-nitrosourea-induced mutagenesis screen (6). *Shd* homozygotes died at birth. Due to the uncontrolled proliferation of basal progenitor cells and a failure of their progeny to differentiate into mature epidermal cells, the *shd* mouse fetuses developed a thick, taut and hyperplastic epidermis with impaired barrier function (6). Later, mutations in KDF1 gene have also been reported in patients with ectodermal dysplasia, affecting the development of eyebrows, teeth, nails, sweat glands and other organs derived from ectoderm (7–9). Evidence from these studies substantiated that KDF1 is a negative regulator of keratinocyte proliferation during epidermis development and an essential promoter for the differentiation of epidermal progenitor cell progeny. Given the key role of KDF1 in the maintenance of the appropriate balance between cell division and differentiation, which is critical for tissue homeostasis and cancer prevention, we speculated that defect in KDF1 might also play a role in the pathogenesis of cancer. To test this idea, in the present study, we examined the expression of KDF1 in the tumor tissue of ccRCC patients in comparison with clinicopathological parameters. Also, we evaluated the effect of altered expression of KDF1 on the phenotype of ccRCC cells.

## Materials and Methods

### Patients

The present study included two cohorts of patients: the TCGA cohort and the TZYY cohort. The TCGA cohort included 530 ccRCC patients, including 344 males and 186 females with a median age of 61 years (ranging from 26 to 90 years) at surgery. The RNA sequencing data of tumor tissue and clinicopathologic data for each patient were downloaded from TCGA database (TCGA-KIRC). The RNA sequencing data of 72 normal renal samples were also downloaded from TCGA database and were used as normal controls. The TZYY cohort included 241 ccRCC patients, including 157 males and 84 females with a median age of 59 years (ranging from 28 to 84 years) at surgery. The patients were hospitalized at Department of Urology, Taizhou Hospital, Wenzhou Medical University from 2004 to 2018 and were histologically confirmed ccRCC after partial or radical nephrectomy. All the patients had no other malignancy history and no history of anticancer therapy before surgery. Patients with mixed histological types were excluded. The clinical and pathological data of TZYY cohort patients were collected from medical records and follow-up records. Here, we defined the overall survival time (OS) as the time interval between surgery and the date of death or the last visit, and the disease-specific survival time (DSS) as the time interval between primary surgery and death from ccRCC or the last follow-up visit. For the analysis of disease-specific mortality, deaths as a result of other causes were censored. In the analysis of immunohistochemistry, 39 non-tumor tissue samples were used as controls. The informed consent has been obtained from all the participants. All research work with human participants was in accordance with the ethical standards of the responsible committee on human experimentation and with the Declaration of Helsinki. The present study was approved by the Ethics Committee of Taizhou Hospital (No. K20200821).

### Immunohistochemical Staining and Analysis

Immunohistochemical staining was performed on formalin-fixed Paraffin sections. Briefly, the sections were deparaffinised in xylene, rehydrated with graded ethanols, autoclaved for antigen repair and treated with 3% hydrogen peroxide solution to inactivate the endogenous peroxidase. After blocking for 30 min in 10% fetal calf serum and rinsed in PBS, the sections were incubated overnight at 4°C with the first antibody, such as rabbit anti-human KDF1 antibody (cat.no. PA5-55926, Invitrogen, MA, USA, dilution 1:200) and rabbit anti-human ki-67 antibody (cat.no. 790-4286, Roche, AZ, USA, dilution 1:2). Then, the sections were washed three times, incubated with the second antibody for 30 min, washed again and developed with diaminobenzidine. Finally, each section was counterstained with haematoxylin. Normal homologous serum was used to replace the first antibody as a negative control. According to the immunostaining intensity, the level of KDF1 was scored by two experienced pathologists in a blind manner: 0, negative; 1, weak; 2, medium; 3, strong. The slides with different score obtained by the two pathologists were reviewed again until the agreed score was made.

For the evaluation of the ratio of ki-67 positive cells in the tumor tissues, at least 15 pictures were taken from each section. The number of ki-67 positive nucleus and total nucleus in each picture were counted and the ratio of ki-67 positive nucleus was calculated. The average value of all the pictures from a section was used as the ki-67 positive ratio of the section.

### Cell Culture, Transduction and Treatment

The ccRCC cell lines 786-O and ACHN were obtained from the Cell Bank of the Chinese Academy of Science (Shanghai, China). The cells were cultured in 1640 medium supplemented with 10% fetal bovine serum. In order to obtain ccRCC cells stably over-expressing KDF1, the cells were transduced with a recombinant lentivirus Lenti-KDF1. Lenti-KDF1 was made by inserting KDF1 coding sequence (152-1348 of NM_152365.3) into the NotI/XbaI site of the lentivirus expression vector pCDH-CMV-MCS-EF1. Infection of ccRCC cells was performed when the cells reached about 50% confluence. Stably transduced cells were obtained by screening the cells with 5μg/ml of puromycin, and the overexpression of KDF1 in the cells was verified by RT-PCR and Western blot.

Lentivirus-mediated short hairpin (sh) RNA was employed to inhibit the expression of KDF1 in KDF1-overexpression cells. To produce the KDF1 shRNA expression Lentivirus Lenti-KDF1shRNA, a shRNA targeting KDF1 was synthesized and cloned into the BamH I and EcoR I site of pLVshRNA-EGFP(2A)puro. Following is the shRNA sequence: 5’-GAGGAGTACTATTCTTTCCATCTCGAGATGGAAAGAATAGTACTCCTCTTTTTT-3’.

### RNA Isolation and RT-PCR Analysis

According to the manufacturer’s protocol, total cellular RNA was isolated with TRIzol^®^ reagent (cat.no. 9109, Thermo Fisher Scientific, MA, USA.) and cDNA was generated using a PrimeScript TM RT Master Mix kit (cat.no. RR01AM, Takara Biotechnology, Dalian, China). A fragment of cDNA was amplified by using the following primers: KDF1 forward, 5’-GTACCCAGCAAGCCATGA-3’ and KDF1 reverse, 5’-CTCCCAGAAAGGGTGTGG-3’.

### Western Blot Analysis

Total protein was extracted using RIPA lysis buffer (cat.no. R0020, Solarbio Technology, Beijing, China) containing Phosphatase Inhibitor and Protease Inhibitor Cocktail (cat.no. C0001,MCE HY-K0023, Targetmol, MA, USA) at 4°C. About 10 µg of total protein was separated by 10% SDS-PAGE and transferred to a PVDF membrane (cat.no. ISEQ00010, Merck KGaA, Darmstadt, Germany). After blocking in the blocking buffer containing 5% skimmed milk powder, the membranes were incubated with the rabbit anti-human KDF1 antibody (cat.no. PA5-55926, Thermo Fisher Scientific, MA, USA, dilution 1:1000) or mice anti-human GAPDH antibody (cat.no. YM3029, Immunoway, TX,USA, dilution 1:1000), overnight at 4°C. Then the membrane was washed and incubated with HRP-conjugated goat anti-rabbit (cat.no. B0201, Immunoway, TX, USA) or rabbit anti-mouse (cat.no. B0101, Immunoway, TX, USA) antibody for 2 h at 37°C. The immunolabeled proteins were detected by chemiluminescence using the Chemiluminescent hRP substrate (Merck KGaA, Darmstadt, Germany). Densitometric analysis was performed using the 1.52a version Image J software (National Institutes of Health, MD, USA).

### Cell Proliferation Assay

A Cell Counting Kit-8 kit (Beyotime Institute of Biotechnology, Shanghai, China) was used in the analysis of cell proliferation according to the manufacturer’s instruction. Briefly, 786-O or ACHN cells were seeded into the 96-well plates at a density of 3×10^3^/well and 5×10^3^/well, respectively. After 6, 24, 48 and 72 hours, the cells were detected by the Cell Counting Kit-8 kit. Here, the results of 6 h were used as a baseline.

### Cell Migration Assay

A wound healing method was used to evaluate the migration of ccRCC cells. Briefly, when the cells grew to confluence, the cells were treated with serum free medium for 24 h. Then a scratch on the cell monolayer was made with a sterile pipette tip. The detached cells were removed by washing with PBS and then the cells were maintained in serum-free RPMI-1640 medium. At regular time, the images of the culture were captured, the un-healing area of each scratch was measured and the wound healing ratio was calculated. The experiments were performed in triplicate.

### Cell Invasion Assay

The relative invasion ability was measured by using BioCoat Matrigel invasion chambers (24-well plates, 8 μm pores, BD Biosciences, CA, USA). Cells were starved by serum free medium for 24 h prior to invasion assays. Then, 5×10^4^ ACHN or 1.5×10^4^ 786-O cells with 200 μL serum-free medium were added to the upper chamber following 500 μL of medium containing 10% FBS being added to the lower chambers. Twenty-four hours later, the cells on the upperside of the membrane were erased and the cells on the downside of the membrane,which have passed through the matrigel and membrane,were fixed with 4% paraformaldehyde, stained with 0.5% crystal violet and counted under Microscope.

### Subcutaneous Xenografts

Four-week old male nude mice were divided randomly into three groups: untransduced cell group, control virus transduced cell group and KDF1 overexpression cell group. Six mice were used in each group. When the untransduced ACHN cells, control virus transduced ACHN cells and KDF1 overexpression ACHN cells grew to nearly 80% confluence, the cells were collected and resuspended in serum-free 1640 medium containing 50% Basement Membrane Extract (cat.no. 2446ML0005, Biofroxx, Einhausen, Germany) at a density of 2×10^7^/ml. Then, for each mouse, 0.1 ml of respective cells (untransduced ACHN cells for the untransduced cell group, control virus transduced ACHN cells for the control virus transduced cell group, and KDF1 overexpression ACHN cells for the KDF1 overexpression cell group) was transplanted subcutaneously on the side of the body. Six weeks later, the mice were euthanized and the tumors were removed, measured and weighed. All experiments were monitored by the Animal Care Committee of Taizhou Hospital and were performed according to the guidelines of the Animal Care Committee of Taizhou Hospital (No.tyz-2020182). All efforts were made to minimize the number of animals used and their suffering.

### Statistical Analysis

SPSS software (version no. 17.0, IBM, CHI, USA) was used to analyze the data. Multiple comparisons were performed using one-way ANOVA. Independent sample T test was used to compare the difference of KDF1 mRNA expression level between the tumor tissue of ccRCC patients and that of the normal controls. Other differences between two groups in the present study were compared by using Mann-Whitney U test. The Spearman correlation analysis was used to explore the correlation of KDF1 mRNA and protein level (represented by score) with the clinicopathological indices. The Kaplan–Meier method and log-rank test were used for survival analysis. The Cox proportional hazards model was used to determine which variables influenced survival. The variables that significantly impacted survival in univariate analyses were included in multivariate analyses. All statistical tests were two tailed, and P values <0.05 were considered significant.

## Results

### Results of TCGA Data Analysis

**Figure 1:**
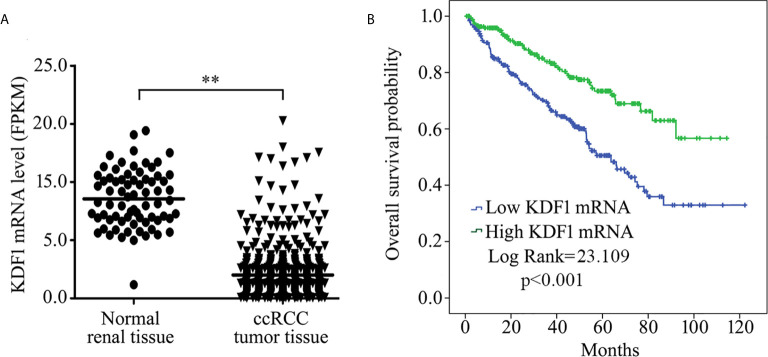
Expression of KDF1 mRNA in the tumor tissue of patients with clear cell renal cell carcinoma (ccRCC) and its association with overall survival. The expression level of KDF1 mRNA in the tumor tissue of 530 ccRCC patients was compared with that in the 72 normal renal samples **(A)**. Patients were divided into higher KDF1 mRNA subgroup (with the KDF1 mRNA level >1.415 Fragments Per Kilobase per Million (FPKM)) and lower KDF1 mRNA subgroup (with the KDF1 mRNA level ≤1.415 FPKM) according to the level of KDF1 mRNA in the tumor tissue and overall survival were compared between the two subgroups by using Kaplan–Meier method **(B)**. In the analysis of overall survival, 23 patients who died within a month after operation were excluded and a total of 507 patients were included. **p < 0.01. FPKM, Fragments per kilobase Million.

We first analyzed the expression of KDF1 mRNA in the tumor tissue of ccRCC patients by using the data from TCGA database. As shown in **Figure 1A**, the expression level of KDF1 mRNA in the tumor tissue was significantly lower than that in normal renal tissue (8.57 ± 2.45 vs 2.00 ± 2.14 Fragments Per Kilobase per Million (FPKM), p<0.01). Analysis based on Speaman coefficient revealed that the expression level of KDF1 mRNA was negatively correlated with the tumor stage (r=-0.221, p=0.0000003, n=530) and Fuhrman grade (r=-0.249, p=0.000000008, n=522).

To determine the association of KDF1 mRNA level with the survival, patients were divided into two groups: high KDF1 mRNA group and low KDF1 mRNA group according to the cutpoint which was evaluated by using EvaluateCutpoints (http://wnbikp.umed.lodz.pl/Evaluate-Cutpoints/). Analysis based on Kaplan-Meier survival revealed that patients with higher KDF1 mRNA had a longer overall survival time than patients with lower KDF1 mRNA **(** **Figure 1B** **)**.

### Expression of KDF1 Protein in the Tumor Tissue of ccRCC Patients Detected by Immunohistochemistry and Western Blot

**Figure 2:**
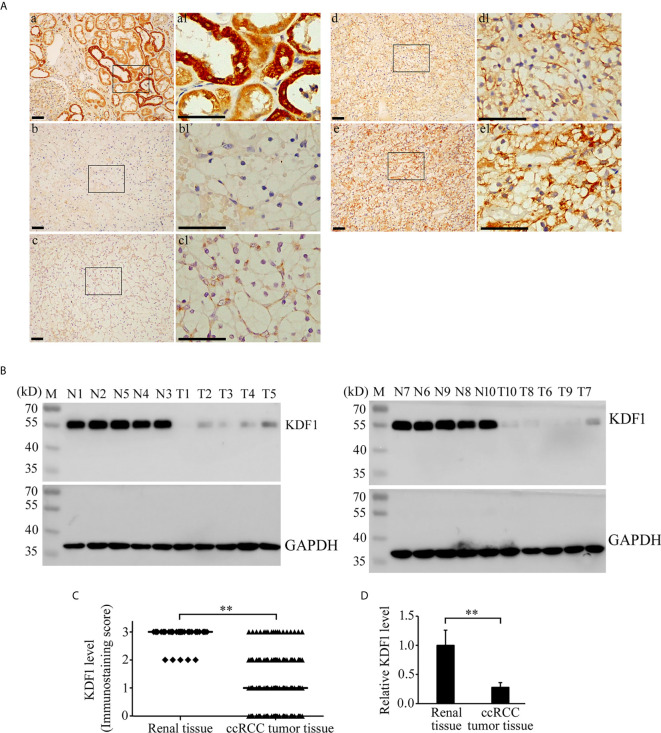
Expression of KDF1 in the tumor tissue of patients with clear cell renal cell carcinoma (ccRCC). Immunohistochemical staining was performed on the tumor tissue of 241 ccRCC patients while 39 non-tumor renal tissue samples were used as controls. The expression of KDF1 in the tumor tissue of 10 ccRCC patients was confirmed by Western blot while 10 non-tumor renal tissues were used as controls. **(A)** Representative pictures of immunohistochemistry. a: A representative picture from non-tumor renal tissue; b: A representative picture from ccRCC patients showing negative immunostaining for KDF1 (the KDF1 level was scored as 0); c: A representative picture from ccRCC patients showing weak immunostaining for KDF1 (the KDF1 level was scored as 1); d: A representative picture from ccRCC patients showing medium immunostaining for KDF1 (the KDF1 level was scored as 2); e: A representative picture from ccRCC patients showing strong immunostaining for KDF1 (the KDF1 level was scored as 3). **(B)** Results of Western blot analysis for KDF1 in the tumor tissue of 10 ccRCC patients and the matched non-tumor tissue. **(C)** Comparison of the KDF1 protein level between the tumor tissue of ccRCC patients and the non-tumor renal tissue according to the results of immunohistochemistry.** (D)** Quantitative analysis of the KDF1 protein level in the tumor tissue of 10 ccRCC patients compared with the non-tumor renal tissue according to the results of Western blot. a1-e1 is the local amplification of a-e respectively. N1-N10: Non-tumor tissue; T1-T10: ccRCC tumor tissue. **P < 0.01. Scale bar: 50 μm.

Immunohistochemical staining was performed on the tumor tissue of 241 ccRCC patients while 39 non-tumor renal tissue samples were used as controls. As shown in **Figure 2A**, KDF1 was extensively expressed in the renal tissue, especially in renal tubular epithelial cells. KDF1 was distributed mainly in a cytoplasmic pattern. Compared with the normal renal tissue, the expression level of KDF1 in the tumor tissue of ccRCC patients decreased markedly. Immunostaining for KDF1 was observed mainly in the cytoplasm of cancer cells.

Among the 241 ccRCC patients, 53, 92, 76 and 20 patients were scored as 0, 1, 2 and 3 respectively according to the immunostaining intensity of KDF1. In contrast, among the 39 non-tumor renal tissue samples, 34 were scored as 3 and 5 were scored as 2 **(** **Figure 2C** **)**.

To confirm the results of immunohistochemistry, the expression of KDF1 in the tumor tissue of 10 ccRCC patients and the corresponding non-tumor tissues was further analyzed by Western blot. As shown in **Figures 2B, D**, results of Western blot analysis indeed showed the decreased expression of KDF1 in ccRCC tumor tissue compared with the non-tumor renal tissue (1 ± 0.25 vs 0.28 ± 0.08, p<0.01).

### Association of KDF1 Protein Level With Clinicopathological Parameters

The association between the expression level of KDF1 protein in the tumor tissue of ccRCC patients and patient’s clinicopathological parameters was analyzed based on the score of immunostaining intensity for KDF1. As shown in **Table 1**, higher expression level of KDF1 protein was observed in patients with high tumor grade. No significant difference in the expression level of KDF1 protein was observed in patients with different ages, gender, location of tumor, tumor size, tumor stage and the habit of smoking and drinking. Also, we did not find significant influence in the expression of KDF1 protein by the presence of necrosis observed in the tumor tissue samples or the presence of hypertension and diabetes mellitus **(** **Table 1** **)**. Analysis based on Spearman coefficient revealed that the expression level of KDF1 protein was correlated negatively with the Fuhrman grade (r=-0.215, p=0.001, n=241).

**Table 1:** Association between KDF1 protein expression level and clinicopathological parameters.

Variable	KDF1 expression level in tumor cells (score)				p
	0	1	2	3	
**Age (years)**					
<60 number (%)	20 (15.6)	49 (38.3)	49 (38.3)	10 (7.8)	0.210
≥60 number (%)	25 (21.9)	43 (37.7)	38 (33.3)	8 (7.0)	
**Gender**					
Male number (%)	31 (19.7)	60 (38.2)	54 (24.4)	12 (7.6)	0.460
Female number (%)	13 (15.5)	32 (38.1)	33 (39.3)	6 (7.1)	
**Tumor size**					
≤4 cm number (%)	23 (16.1)	56 (39.2)	53 (37.1)	11 (7.7)	0.449
>4 cm number (%)	21 (21.4)	36 (36.7)	34 (34.7)	7 (7.1)	
**Stage**					
1∼2 number (%)	36 (16.9)	87 (40.8)	72 (33.8)	18 (8.5)	0.865
3∼4 number (%)	8 (28.6)	5 (17.9)	15 (53.6)	0 (0.0)	
**Fuhrman grade**					
1-2 number (%)	28(15.1)	67 (36.0)	73 (39.2)	18 (9.7)	0.0004
3-4 number (%)	16 (29.1)	25 (45.5)	14 (25.5)	0 (0.0)	
**Location**					
Left number (%)	25 (22.3)	42(37.5)	35(31.3)	10 (8.9)	0.268
Right number (%)	19(14.7)	50 (38.8)	52 (40.3)	8 (6.2)	
**Smoking**					
Yes number (%)	15 (22.4)	26 (38.8)	22 (32.8)	4 (6.0)	0.250
No number (%)	29 (16.7)	66 (37.9)	65(37.4)	14 (8.0)	
**Drinking**					
Yes number (%)	4 (14.3)	13 (46.4)	11 (29.3)	0 (0.0)	0.683
No number (%)	40 (18.8)	79 (37.1)	76 (35.7)	18 (8.5)	
**Hypertension**					
Yes number (%)	18 (20.0)	38 (42.2)	29 (32.2)	5 (5.6)	0.179
No number (%)	26 (17.2)	54 (35.8)	58 (38.4)	13 (8.6)	
**Diabetes**					
Yes number (%)	3 (11.5)	13 (50.0)	9 (34.6)	1 (3.8)	0.876
No number (%)	41 (18.2)	79 (35.1)	88 (39.1)	17 (7.5)	
**Necrosis in tumor**					
Yes number (%)	5 (13.2)	13 (34.2)	18 (47.4)	2 (5.3)	0.287
No number (%)	39 (19.2)	79 (38.9)	69 (34.0)	16 (7.9)	

Number, the number of patients.

### Results of Survival Analysis Based on KDF1 Protein Level in the Tumor Tissue of ccRCC Patients

**Figure 3:**
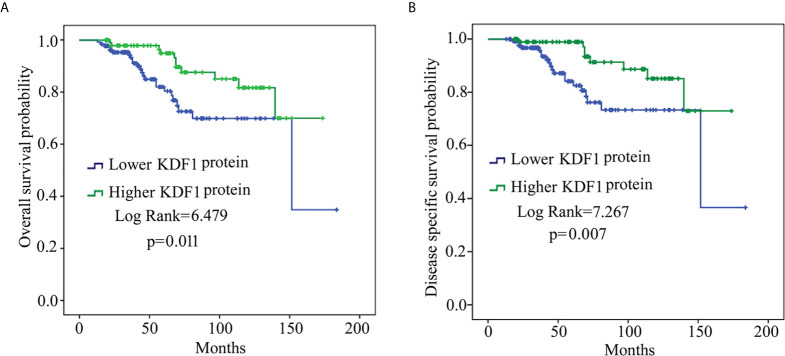
Results of survival analysis of patients with clear cell renal cell carcinoma (ccRCC) based on KDF1 protein level in the tumor tissue. A total of 241 ccRCC patients were included. The patients were divided into lower KDF1 protein subgroup (with immunostaining score for KDF1 in the tumor tissue being 0 or 1, n=136) and higher KDF1 protein subgroup (with immunostaining score for KDF1 in the tumor tissue being 2 or 3, n=105) according to the level of KDF1 protein in the tumor tissue of the patients. The overall survival **(A)** and disease-specific survival **(B)** were compared between the two subgroups by using Kaplan-Meier method.

To determine whether the expression level of KDF1 is associated with the survival, patients were divided into lower KDF1 protein group (including patients of score 0 and 1) and higher KDF1 protein group (including patients of score 2 and 3) according to the immunostaining intensity for KDF1 in the tumor tissue and a survival analysis using Kaplan-Meier method was performed. As shown in **Figure 3**, patients with higher KDF1 protein level in the tumor tissues were found to have a longer OS and DSS when compared with patients with lower KDF1 protein.

Analysis based on Univariable Cox regression revealed that KDF1 protein level in the tumor tissue of ccRCC patients was associated significantly with both OS and DSS along with the age of the patients, the size of the tumors, Fuhrman grade and tumor stage. Analysis based on Multivariable Cox regression revealed that the level of KDF1 protein in the tumor tissue was not associated significantly with the OS of the patients, but it still had a significant association with the DSS along with the tumor stage and tumor Fuhrman grade **(** **Tables 2** and **3** **)**.

**Table 2:** Univariable and multivariable Cox regression analysis for overall survival.

Variables	Univariate analysis		Multivariate analysis	
	HR (95.0% CI)	p value	HR (95.0% CI)	p value
Age	3.15 (1.51-6.58)	0.002	2.15 (1.00-4.65)	0.051
≤59 vs >59 (years)				
Stage	3.82 (1.92-7.61)	0.0001	2.25 (1.06-4.79)	0.036
1∼2 vs 3∼4				
Gender	0.98 (0.48-2.01)	0.954		
Male vs female				
Tumor size	2.05 (1.04-4.04)	0.038	1.41 (0.68-2.91)	0.356
≤4 vs >4 (cm)				
Fuhrman Grade	5.17 (2.60-10.29)	0.000003	3.17 (1.48-6.81)	0.003
1∼2 vs 3∼4				
KDF1 level	0.40 (0.20-0.83)	0.014	0.52 (0.25-1.10)	0.085
Low vs high				
Hypertension	0.99 (0.49-1.97)	0.968		
Yes vs no				
Diabetes Mellitus	0.85 (0.26-2.78)	0.786		
Yes vs no				

HR, hazard ratio; CI, confidence interval.

**Table 3:** Univariable and multivariable Cox regression analysis for Disease specific survival.

Variables	Univariate analysis		Multivariate analysis	
	HR (95.0% CI)	p value	HR (95.0% CI)	p value
Age	2.26 (1.04-4.92)	0.04	1.11 (0.47-2.65)	0.807
≤59 vs >59 (years)				
Stage	5.49 (2.60-11.59)	0.000008	2.55 (1.12-5.81)	0.026
1∼2 vs 3∼4				
Gender	0.92 (0.41-2.05)	0.840		
Male vs female				
Tumor size	3.38 (1.49-7.69)	0.004	2.16 (0.92-5.07)	0.076
≤4 vs >4 (cm)				
Fuhrman Grade	9.14 (3.87-21.55)	0.0000004	5.77 (2.32-14.31)	0.0002
1∼2 vs 3∼4				
KDF1 level	0.34 (0.15-0.77)	0.010	0.42 (0.18-0.97)	0.041
Low vs high				
Hypertension	0.91 (0.41-1.99)	0.806		
Yes vs no				
Diabetes Mellitus	1.11 (0.33-3.68)	0.868		
Yes vs no				

HR, hazard ratio; CI, confidence interval.

### Effect of Overexpression of KDF1 on the Proliferation, Migration and Invasion of ccRCC Cells

**Figure 4:**
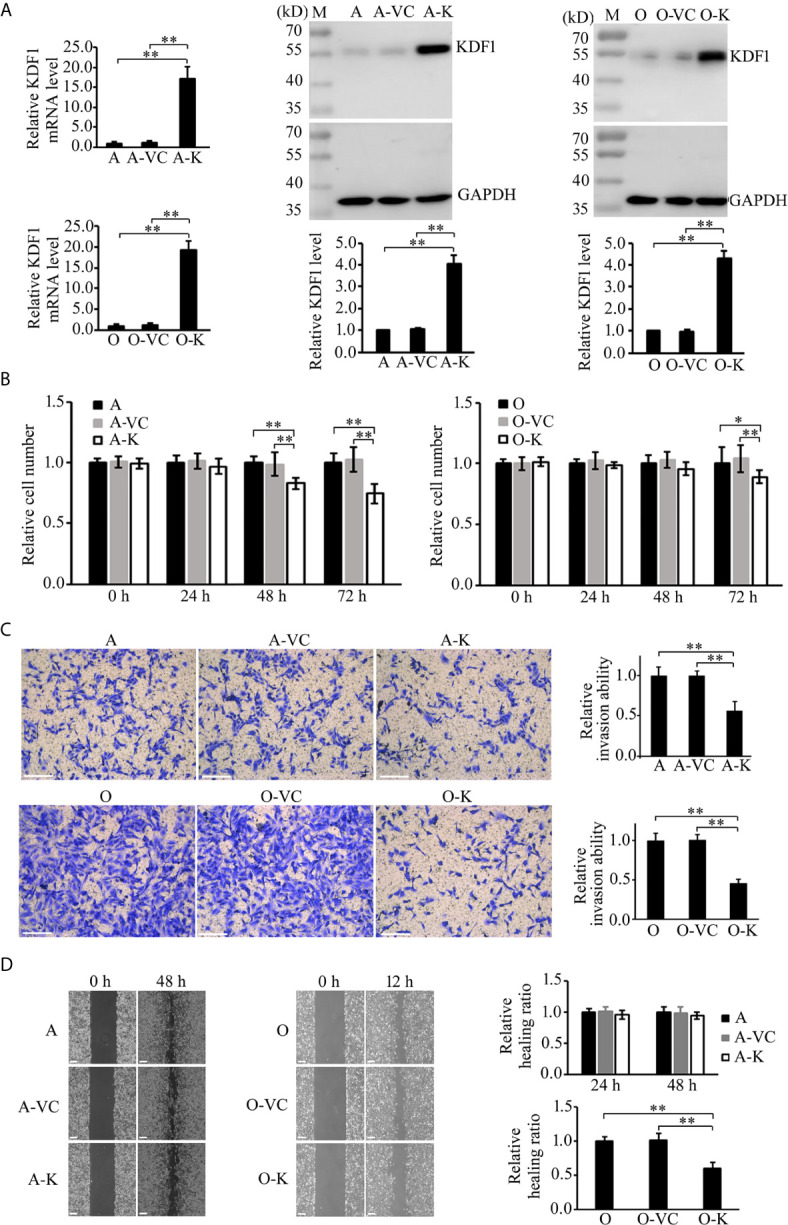
Effect of KDF1 overexpression on the proliferation, migration and invasion of ccRCC cells. Two ccRCC cell lines, 786-O and ACHN, were used in the experiments. The KDF1 overexpression ccRCC cells, 786-O-KDF1 and ACHN-KDF1, were constructed *via* stably infecting 786-O and ACHN cells with a recombinant KDF1 expression lentivirus. The overexpression of KDF1 in ccRCC cells were confirmed by quantitative RT-PCR and Western blot **(A)** and the influence of KDF1 overexpression in the proliferation **(B)**, migration **(C)** and invasion **(D)** of the ccRCC cells were evaluated by using CCK-8, wound healing and Matrigel invasion chamber methods. All the experiments were repeated at least three times. A, untransduced ACHN cells; A-VC, control virus transduced ACHN cells; A-K, KDF1 overexpression ACHN cells; O, untransduced 786-O cells; O-VC, control virus transduced 786-O cells; O-K, KDF1 overexpression 786-O cells. *p < 0.05; **p < 0.01. Scale bar, 100µm.

To determine the possible role of KDF1 in ccRCC cells, we first examined the effect of KDF1 overexpression on the phenotype of ACHN and 786-O cells. KDF1 over-expressing ccRCC cells ACHN-KDF1 and 786-O-KDF1 were established through stably transducing ACHN and 786-O cells with the recombinant lentivirus Lenti-KDF1. The increased expression of KDF1 mRNA (16.75 ± 2.98 vs 1 ± 0.24 and 1.19 ± 0.30 in ACHN cells, p<0.01; 18.72 ± 1.96 vs 1 ± 0.30 and 1.14 ± 0.37 in 786-O cells, p<0.01) and protein (4.06 ± 0.41 vs 1 and 1.04 ± 0.07 in ACHN cells, p<0.01; 4.27 ± 0.36 vs 1 and 0.98 ± 0.05 in 786-O cells, p<0.01) in the cells was proved by quantitative RT-PCR and Western blot (**Figure 4A**). As shown in **Figure 4**, overexpression of KDF1 significantly inhibited the proliferation (0.74 ± 0.08 vs 1 ± 0.07 and 1.03 ± 0.10 in ACHN cells at 72h, p<0.01; 0.89 ± 0.05 vs 1.0 ± 0.14 and 1.04 ± 0.11 in 786-O cells at 72h, p<0.05, **Figure 4B**) and invasion (0.56 ± 0.12 vs 1 ± 0.11 and 0.99 ± 0.06 in ACHN cells, p<0.01; 0.46 ± 0.05 vs 1 ± 0.10 and 1.01 ± 0.07 in 786-O cells, p<0.01, respectively, **Figure 4C**) of ACHN and 786-O cells. However, significantly decreased migration was only observed in 786-O cells (0.60 ± 0.08 vs 1 ± 0.06 and 1.02 ± 0.09, p<0.01, **Figure 4D**).

### Overexpression of KDF1 Significantly Decreased the Growth of Xenograft Tumors Produced by ACHN Cells

**Figure 5:**
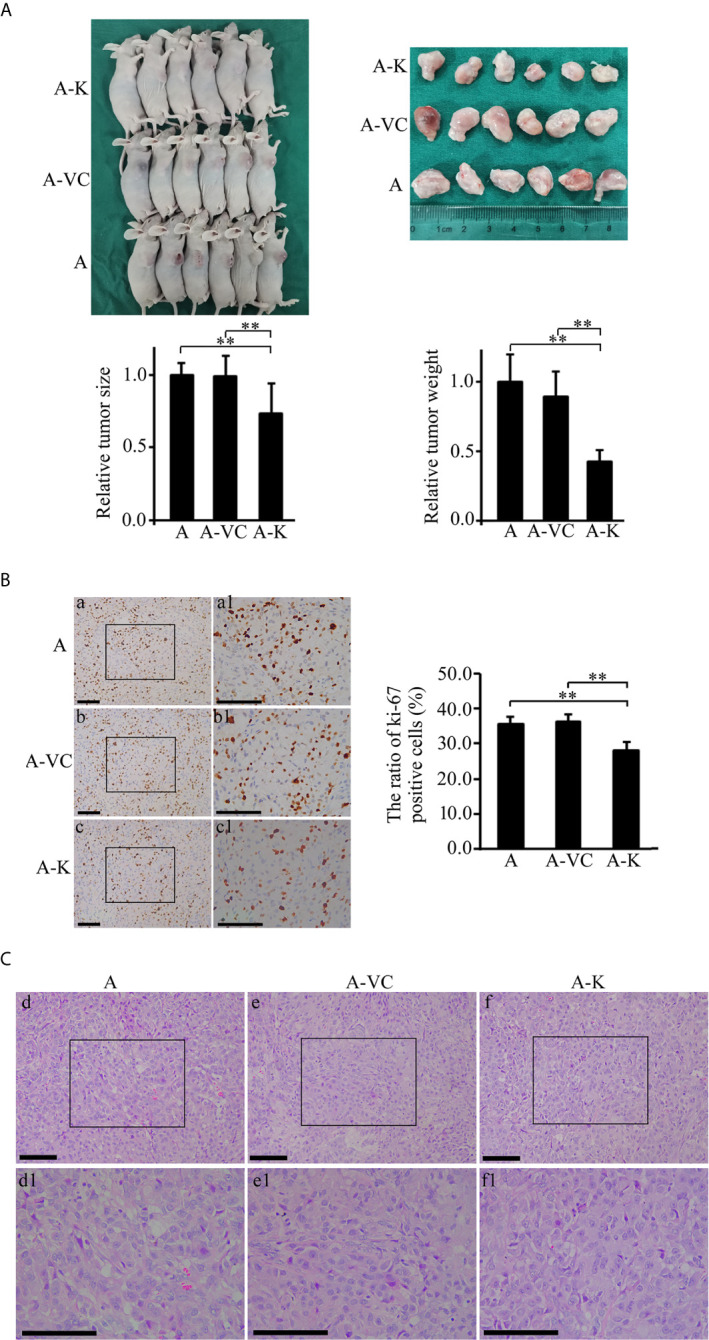
Overexpression of KDF1 significantly decreased the growth of transgrafted tumors and the ratio of ki-67 positive cells in the tumor. Four-week old male nude mice were randomly divided into non-transduced cell group, control virus transduced cell group and KDF1 overexpression cell group. Six mice were used in each group. For each mouse, 2×10^6^ cells (untransduced ACHN cells for the untransduced cell group, control virus transduced ACHN cells for the control virus transduced cell group, and KDF1 overexpression ACHN cells for the KDF1 overexpression cell group) were transplanted subcutaneously on the side of the body. Six weeks later, the mice were euthanized and the tumors were removed, measured and weighed. Paraffin sections of the transgrafted tumors were used in Hematoxylin-Eosin (HE) staining and immunohistochemical staining for ki-67. Figure part **(A)** Results of the tumor transplant trial showing that overexpression of KDF1 decreased the growth of transgrafted tumors. Figure part **(B)** Results of immunohistochemical staining for ki-67 showing that overexpression of KDF1 decreased the ratio of ki-67 positive cells in the transgrafted tumors. Figure part **(C)** Results of HE staining showing no structural difference among the tumor tissues. A, the untransduced cell group; A-VC, the control virus transduced cell group; A-K, the KDF1 overexpression cell group. a1-f1 is a partial magnification of a-f, respectively. **P < 0.01.

?A3B2 twb 0.24w?>Given that overexpression of KDF1 was found to decrease the proliferation of ccRCC cells *in vitro*, we suppose that overexpression of KDF1 might also reduce the growth of ccRCC tumor. To test this possibility, a xenograft trial was performed by using the untransduced, control virus transduced and KDF1 over-expressing ACHN cells. As shown in **Figure 5A**, ACHN cells over-expressing KDF1 produced much smaller xenograft tumors compared with those produced by the control cells (0.73 ± 0.21 vs 1 ± 0.08 and 0.99 ± 0.14 in size, p<0.01; 0.43 ± 0.08 vs 1 ± 0.20 and 0.89 ± 0.18 in weight, p<0.01). To determine whether the decreased tumors were caused by the decreased proliferation of ccRCC cells, we further examined the expression of ki-67, a molecular marker for proliferation cells, in the tumor tissues. As shown in **Figure 5B**, the percentage of ki-67 positive cells in the tumors derived from the KDF1 overexpression cells was significantly lower compared with that in tumors derived from the control cells (28.11 ± 2.41 vs 35.59 ± 1.91 and 36.32 ± 1.93, p<0.01). However, we did not observed difference in the structure of the tumors (**Figure 5C**).

### Knock-Down of KDF1 in KDF1 Over-Expressing Cells Restore the Phenotype of ccRCC Cells

**Figure 6:**
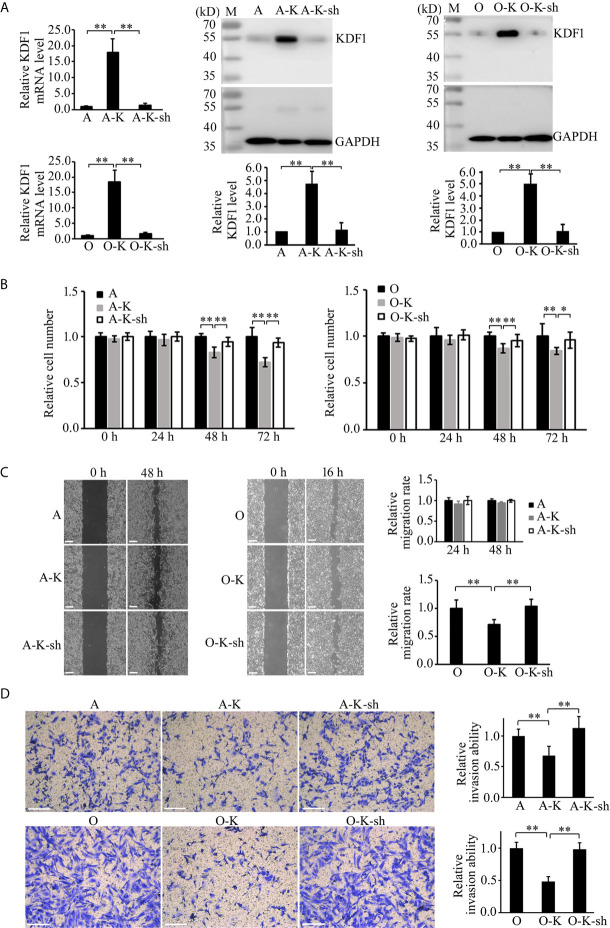
Knockdown of KDF1 reversed the effect of KDF1 overexpression on the ccRCC cell’s proliferation, migration and invasion. A recombinant KDF1 shRNA expression lentivirus was used to knock down the expression of KDF1 in the KDF1 overexpression ccRCC cells. The knockdown of KDF1 expression was confirmed by quantitative RT-PCR and Western blot analysis **(A)** and the influence of KDF1 knockdown in the proliferation **(B)**, migration **(C)** and invasion **(D)** of the KDF1 overexpression ccRCC cells were evaluated by using CCK-8, wound healing and Matrigel invasion chamber methods. All the experiments were repeated at least three times. A, untransduced ACHN cells; A-K, KDF1 overexpression ACHN cells; A-K-sh, the KDF1 knockdown A-K; O, untransduced 786-O cells; O-K, KDF1 overexpression 786-O cells; O-K-sh, the KDF1 knockdown O-K; *p < 0.05; **p < 0.01. Scale bar, 100µm.

To determine whether the phenotypic changes in KDF1 overexpressing ccRCC cells is caused by the increased KDF1 in the cells, we knocked down the expression of KDF1 in the KDF1 overexpressing cells by transducing them with a shRNA overexpression recombinant lentivirus, which was designed to express a shRNA targeting KDF1. As shown in **Figure 6A**, transduction of the KDF1 over-expressing cells 786-O-KDF1 and ACHN-KDF1 with the lentivirus significantly reduced the expression of KDF1 mRNA (1.47 ± 0.54, 18.22 ± 3.57 and 1 ± 0.22 in the knockdown, KDF1 overexpression and untransduced 786-O cells; 1.41 ± 0.57, 17.71 ± 4.16 and 1 ± 0.21 in the knockdown, KDF1 overexpression and untransduced ACHN cells) and protein (1.06 ± 0.58, 5.02 ± 0.84 and 1 in the knockdown, KDF1 overexpression and untransduced 786-O cells; 1.11 ± 0.56, 4.69 ± 1.00, and 1 in the knockdown, KDF1 overexpression and untransduced ACHN cells) in these cells. In the meanwhile, it markedly reversed the ccRCC cells’ inhibition in the proliferation (0.96 ± 0.09, 0.84 ± 0.04 and 1 ± 0.13 in the knockdown, KDF1 overexpression and untransduced 786-O cells at 72 h; 0.94 ± 0.05, 0.72 ± 0.05 and 1 ± 0.10 in the knockdown, KDF1 overexpression and untransduced ACHN cells at 72 h, **Figure 6B**), migration **(**1.04 ± 0.13, 0.72 ± 0.08 and 1 ± 0.15 in the knockdown, KDF1 overexpression and untransduced 786-O cells; 1.01 ± 0.03, 0.94 ± 0.03 and 1 ± 0.04 in the knockdown, KDF1 overexpression and untransduced ACHN cells, **Figure 6C** **)** and invasion **(**0.98 ± 0.10, 0.48 ± 0.08 and 1 ± 0.09 in the knockdown, KDF1 overexpression and untransduced 786-O cells; 1.13 ± 0.20, 0.67 ± 0.16 and 1 ± 0.11 in the knockdown, KDF1 overexpression and untransduced ACHN cells, **Figure 6D** **)** caused by KDF1 overexpression.

## Discussion

In the present study, we examined the expression of KDF1 in the tumor tissue of ccRCC patients using two cohorts of patients and compared it with the clinicopathological indices of the patients. Analysis based on RNA sequencing data from TCGA database showed that the expression level of KDF1 mRNA decreased markedly in the tumor tissues of ccRCC patients compared with that in the normal renal tissues. The expression level of KDF1 mRNA was found to correlate negatively with tumor grade and tumor stage, and positively with patients’ OS. In accordance with the results of mRNA expression, KDF1 protein was also found to be down-regulated in the tumor tissues of ccRCC patients compared with that in the normal renal tissues. The decreased expression of KDF1 in the tumor tissue was further confirmed by Western blot analysis. The level of KDF1 protein in the cancer cells was found to correlate negatively with tumor grade. Patients of ccRCC with higher KDF1 protein in cancer cells were found to have longer OS and DSS and KDF1 was demonstrated to be an independent factor associated with patients’ DSS. Based on the above results, we believe that patients with higher KDF1 expression tend to have better prognosis compared with those with lower KDF1 expression. It is believed that renal cell carcinoma including ccRCC is derived from renal tubular epithelial cells. The present clinical finding suggested that down-regulation of KDF1 might be involved in the pathogenesis of ccRCC and KDF1 might function as a tumor suppressor. In support of this idea, in the present study, overexpression of KDF1 was observed to decrease the proliferation, migration and invasion of ccRCC cells, which could be reversed by knocking down the expression of KDF1 in the cells. Also, KDF1 over-expressing ccRCC cells were found to produce significantly smaller tumors in the xenograft tests. Furthermore, decreased ki-67 positive cells were observed in the xenograft tumor tissue derived from the KDF1 overexpression ccRCC cells compared with those xenograft tumors derived from the control ccRCC cells. To our knowledge, this is the first report on the expression and role of KDF1 in ccRCC.

KDF1 was first reported by Lee and his colleagues in a forward genetic study, in which KDF1 was demonstrated to play a key role in the development of normal epidermis through regulating the proliferation and differentiation of keratinocytes (6). Since then, several other studies have reported the involvement of KDF1 in ectodermal organ development (7–9). In particular, mutation of KDF1 has been reported to be associated with tooth agenesis (7, 9). Interestingly, there is an emerging debate about the connection of tooth agenesis to cancer: On the one hand, some family studies indicated that presence of tooth agenesis meant higher incidence of some cancers including colorectal cancer and epithelial ovarian cancer, but on the other hand, some case-controlled molecular studies showed that there was no significant association between tooth agenesis and the occurrence of these cancer (10). Given the common signaling pathways shared in tooth development and tumorigenesis (10), the molecular abnormity that caused tooth agenesis may also lead to tumorigenesis. To our knowledge, no study has reported the role of KDF1 in cancer. Thus, the present finding about the involvement of KDF1 in ccRCC not only has broadened the window of understanding the pathological function of KDF1, but has also provided a novel link between tooth agenesis and ccRCC. However, further systematic research should be performed before exact conclusion can be drawn.

As a newly discovered molecule, data about the function of KDF1 is still limited. To make things worse, although it is an evolutionarily conserved protein, there is no homologous experimental structure available that would serve as a good-confidence support for modeling the 3D structure of KDF1 (11). Thus, the functional information deduced from structural bioinformatic analysis about this molecule is quite limited. According to the prediction of secondary structure, KDF1 is unlikely to be an enzyme or transmembrane transporter or receptor, instead, it might function as a protein-binding adaptor, scaffold and/or cofactor (7). Indeed, stratifin was found to interact genetically with KDF1 (6) and in a recent study, KDF1 was found to regulate skin differentiation through deubiquitination and stabilization of IKKa (12). In addition, *shd* mutant was also found opposite in phenotype to a previously mutant caused by p63 loss (6, 13–15) and in the study carried out by Lee et al., reducing the dosage of p63 rescued many aspects of the *shd* phenotype (6), indicating that KDF1 regulates Keratinocyte differentiation through inhibiting the expression of p63. Stratifin, also known as 14-3-3-σ, is a protein member of 14-3-3 family, which has been reported to be involved in a variety of essential cellular functions including cell proliferation, differentiation, survival, apoptosis, and cytoskeletal integrity (16). As a transcription factor of p53 family, p63 has been well-studied and proved to play a crucial role in the regulation of epidermal cell proliferation and differentiation. In addition, the protein has been reported to be involved in the development of many tumors through regulating the expression of its target genes (17). IKKa is key member of NF-κB signaling system. Through regulating the degradation of IκB, the specific inhibitor of NF­κB, IKKα plays a key role in NF­κB based signal transduction, which is important in a variety of biological process including inflammation and tumor development (18). Besides, IKKα has also been reported to exert its roles in an NF­κB signaling-independent way, which is especially important in the pathogenesis of some cancers (19). Of note, the roles of stratifin, p63 and IKK in tumor development have been demonstrated to be cell context-specific. Both tumor suppressive and promotive roles have been reported for these molecules in different cancers (20–22). Therefore, the functional association of KDF1 with stratifin, p63 and IKK might have pointed a road for dissecting the mechanism underlying the pathogenic role of KDF1 in ccRCC. However, more studies are needed to answer this question.

It should be pointed out that there are some differences in the effects of KDF1 overexpression on the phenotype of the two ccRCC cell lines, 786-O and ACHN. While overexpression of KDF1 significantly reduced the migration of 786-O cells, its influence in the migration of ACHN cells is quietly limited, not reaching the significant level. Again, the influence of KDF1 overexpression in the proliferation of 786-O cells was smaller than that in ACHN cells although it still reached the significant level when compared with the control groups. For the present, we don’t know the exact cause for this, but this phenomenon is quite similar to that found in stratifin, p63 and IKK (20–22), three molecules found to be functionally associated with KDF1, and emphasizes the importance of taking the specific cellular context into account when we discuss the function of KDF1.

Limitations are present in the present study. First of all, the population investigated in the present study is not very large, especially in the part of KDF1 protein expression; deviation due to patient selection might be inevitable. Secondly, the present study has not explored the molecular mechanism through which KDF1 exerts its roles. To well dissect the pathologic significance of KDF1, further mechanism research is essential. Thirdly, the present study only used two ccRCC cell lines. Given the fact that the function of KDF1 may be context-specific, it is better to examine the roles of this molecule in more ccRCC cell lines.

In summary, for the first time, the present study investigated the expression and function of KDF1 in the tumor tissue of ccRCC patients. KDF1 was found to be decreasingly expressed in the cancer cells and correlated negatively with the tumor grade and positively the survival of the patients. Overexpression of KDF1 was shown to reduce the proliferation, migration and invasion of ccRCC cells, which could be reversed by re-knock down of KDF1. Also, overexpression of KDF1 was found to inhibit the growth of xenograft tumors. All these suggest that decreased expression of KDF1 is involved in the pathogenesis of ccRCC and KDF1 may function as a tumor suppressor. Thus, the present study has opened a novel window for understanding the pathological function of KDF1 in ccRCC and thrown a novel beam of light on the pathogenic mechanism of the disease. However, further research is still needed to prove our findings and dissect the function of KDF1 in ccRCC.

## Data Availability Statement

The original contributions presented in the study are included in the article/supplementary material. Further inquiries can be directed to the corresponding author.

## Ethics Statement

The animal study was reviewed and approved by the Animal Care Committee of Taizhou Hospital. All research work concerning human participants was reviewed and approved by the Ethics Committee of Taizhou Hospital.

## Author Contributions

J-mZ designed the study, participated in the experiments and analysis of the data, drafted the manuscript and gave final approval of the version to be published. M-fG and H-yY participated in the design of the study, analysis of the data and revision of the manuscript, and gave final approval of the version to be published. L-xY, H-xZ, J-qB, and Y-qG participated in the performance of the experiments, analysis of the data, and gave final approval of the version to be published. Q-xY and Y-hX participated in revision of the manuscript, and gave final approval of the version to be published. All authors contributed to the article and approved the submitted version.

## Funding

The project was supported by a Doctor funding from Enze medical center (2017BSKYQDJJ01).

## Conflict of Interest

The authors declare that the research was conducted in the absence of any commercial or financial relationships that could be construed as a potential conflict of interest.
